# Associations Between Early/Current Neighborhood Deprivation and Midlife Cognitive Functioning: Results from the Colorado Adoption/Twin Study of Lifespan behavioral development and cognitive aging (CATSLife)

**DOI:** 10.1002/alz70860_101017

**Published:** 2025-12-23

**Authors:** Elizabeth Muñoz, Lourdes S. Romañach Álvarez, Jean Choi, Robin P Corley, Soo H Rhee, Sally J Wadsworth, Chandra A. Reynolds, The CATSLife Team

**Affiliations:** ^1^ University of Texas at Austin, Austin, TX, USA; ^2^ University of Colorado Boulder, Boulder, CO, USA; ^3^ Institute for Behavioral Genetics, University of Colorado Boulder, Boulder, CO, USA; ^4^ University of California, Riverside, Riverside, CA, USA

## Abstract

**Background:**

Growing evidence highlights the impact of life course environmental contexts on cognitive function. Early‐life and adult neighborhood disadvantage may affect midlife cognitive abilities, a stage when subtle changes can indicate early neurocognitive impairments. We examined the independent and joint associations between early‐life and concurrent neighborhood disadvantage with specific cognitive abilities in midlife.

**Methods:**

We leveraged data from 1,141 CATSLife participants (*Mage* = 33.03, *SD* = 4.84; 53.37% female) with address data in infancy and concurrently in early midlife. Between 2015 and 2021, participants completed an in‐person battery of verbal abilities, memory, spatial abilities, and perceptual speed (higher scores indicated better performance). Addresses at the time of testing were geocoded and linked with 2010 Census data. First address data were matched to the 1980 or 1990 Census and harmonized to 2010 tract boundaries using the Longitudinal Tract Database. An Area Deprivation Index (ADI) score, calculated as deciles and averaged, measured neighborhood disadvantage for both early‐life and current addresses. Generalized Estimating Equations were used to adjust for family clustering and assess the effects of early‐life and current ADI on cognitive abilities, controlling for age, sex, race, adoptive status, and APOE genotype.

**Results:**

Early‐life and current ADI were not associated with memory or verbal abilities. Early‐life ADI (B = ‐0.06, SE = 0.03, *p* = 0.04) and current ADI (B = ‐0.06, SE = 0.03, *p* = 0.03) were each independently associated with poorer processing speed, but neither remained significant when both ADI scores were included in the same model. Current ADI was independently associated with lower spatial functioning, even after adjusting for early‐life ADI (B = ‐0.05, SE = 0.02, *p* = 0.04). Early‐life ADI was not independently associated with spatial functioning. No significant interactions between early‐life and current ADI were observed for any cognitive abilities.

**Conclusion:**

Early‐life and current neighborhood deprivation were independently associated with poorer processing speed. Additionally, current neighborhood deprivation was linked to poorer spatial functioning, even when accounting for early‐life conditions. These results underscore the importance of considering both early and concurrent neighborhood environments as potential influences on cognitive functioning.

